# Intramural Hematoma as Unexpected Complication of COVID-19 Infection

**DOI:** 10.1055/s-0040-1713107

**Published:** 2020-06-04

**Authors:** Francesca Terzi, Mariano Cefarelli, Rossella Fattori, Marco Di Eusanio

**Affiliations:** 1 Cardiology Unit, Azienda Ospedaliera Ospedali Riuniti Marche Nord, Pesaro, Italy; 2 Cardiac Surgery Unit, Lancisi Cardiovascular Center, Polytechnic University of Marche, Ancona, Italy; 3 Cardiovascular Center, Marfan Center, Ospedali Riuniti Marche, Ancona, Italy

**Keywords:** intramural hematoma, COVID-19, aorta, inflammation, cytokine storm

## Abstract

Novel coronavirus disease-2019 (COVID-19) is an ominous infectious disease that seems capable to attack any organ system, leading in the most severe cases to patient death. COVID-19 has been associated with multiple cardiovascular complications of inflammatory and immune origin, leading to a wide spectrum of vascular damage, myocardial injury, stroke, and pulmonary obstruction. We report the case of a patient with COVID-19 infection who developed an acute aortic syndrome with the characteristics of aortic intramural hematoma.

## Introduction

Patients with preexisting cardiovascular disease (CVD) have an increased risk of severe complications from novel coronavirus disease 2019 (COVID-19). However, even COVID-19 patients without a history of CVD may be at risk for cardiovascular complications, 1 2 often constituting the primary cause of death.

Aortic intramural hematoma (IMH) is an important subgroup of acute aortic syndrome (AAS) and may account for 10 to 25% of them. When it involves the ascending aorta, it is highly lethal and requires emergent surgery. 3 Nuclear imaging and biomarker-based studies have suggested a strong association between aortic wall inflammation and IMH, providing information on its risk stratification. 4 5

Here, we present the case of a patient with COVID-19 infection complicated with IMH who underwent emergency surgery.

## Case Presentation

A 73-year-old Italian man, with an unremarkable clinical history and no cardiovascular risk factors, was admitted to the emergency department with chest pain and shortness of breath, along with lower limb paresthesia. The patient disclosed a 10-day history of fever, cough, and sore throat, treated with paracetamol 2 g/day. Based on real-time polymerase chain reaction analysis of a sputum sample, he was diagnosed with COVID-19.

**Fig. 1:**
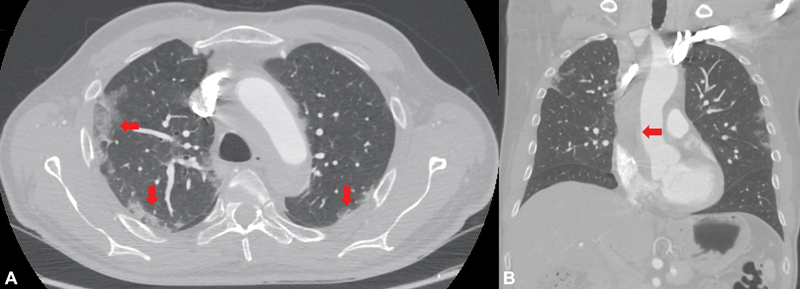
Computed tomography scan of the chest: ( **A** ) multiple ground-glass opacities with bilateral parenchymal consolidation and interlobular septal thickening (red arrows) ( **B** ) intramural hematoma of ascending aorta (red arrow) with periadventitial aortic wall enhancement.

On presentation, he was normotensive; heart rate was 60 bpm and body temperature was 38°C. Laboratory tests demonstrated high inflammatory markers and lymphopenia (white blood cell count, 20.070/mm ^3^ ; neutrophils, 92%; lymphocytes, 1.5%; and C-reactive protein, 20.50 mg/dL), increased high sensitive Troponin I (200 ng/L), and N-terminal pro-brain natriuretic peptide (4,000 pg/mL). Even interleukin (IL)-6 level was elevated (121 pg/mL). Estimated glomerular filtration rate and liver enzymes were unremarkable. Chest contrast-enhanced computed tomography (CT) revealed multiple ground-glass opacities with bilateral parenchymal consolidation and interlobular septal thickening suggestive for COVID-19 interstitial pneumonia ( Fig. 1 ). Moreover, CT showed an IMH extending from the ascending aorta (supravalvular plane) to the abdominal aorta. The aorta was normal in dimension (Valsalva's sinuses, 37 mm; ascending aorta, 36 mm; aortic arch, 30 mm).

The patient was transferred urgently to a referral hospital for cardiac surgery. The aorta and the heart were exposed through a median sternotomy. Under moderate hypothermic circulatory arrest (28°C nasopharyngeal temperature) and antegrade selective cerebral perfusion (ASCP), the ascending aorta and hemiarch were replaced by means of a 28 mm straight vascular graft. cardiopulmonary bypass, cross-clamp, and ASCP times were, 58, 40, and 10 minutes, respectively. All team members were protected with an N95 respirator mask and wore double gowns, gloves, and eye protection.

The patient, extubated on postoperative day (POD) 1, experienced respiratory insufficiency requiring reintubation 3 days later and remained under ventilation for 7 days. Noninvasive ventilation therapy was applied during the 72 hours following reextubation with beneficial effects. For the concomitant COVID-19 disease, the patient received empiric treatment with hydroxychloroquine and lopinavir/ritonavir. Before transferring the patient to the referral hospital on POD 19, the patient transited from the intensive care unit (ICU) through our COVID-19 mid- and low-intensive care units. At discharge, the COVID-19 test was still positive. The patient is now following a rehabilitation program and two consecutive COVID-19 tests are negative.

## Discussion

Cardiovascular involvement has been reported in patients with COVID-19; inflammation in the vascular system can result in diffuse microangiopathy, thrombosis, and vascular damage. 1 Inflammation of the aortic wall also seems to play a role in the pathogenesis of AAS and in particular of IMH. 7 Several previous studies have suggested that inflammation exists in the involved aorta in patients with classic AD 5 6 7 8 and IMH. 7 Our COVID-19 patient without cardiovascular risk factors complicated by IMH could intriguingly indicate IMH as another potential COVID-19 major vascular complication; the devastating systemic inflammatory response to COVID-19 infection with the typical “cytokine storm” could likely represent the underlying mechanism of the aortic wall damage. However, only the evolving understanding of the multiple mechanisms in which the virus, or the immune response to it, attacks human cells will lead to a better comprehension and treatment of the protean manifestations of COVID-19 disease.

## References

[1] Driggin E Madhavan M V Bikdeli B Cardiovascular considerations for patients, health care workers, and health systems during the coronavirus disease 2019 (COVID-19) pandemic J Am Coll Cardiol 2020 75 18 10.1016/j.jacc.2020.03.031 PMC7198856 32201335

[2] Zheng Y Y Ma Y T Zhang J Y Xie X COVID-19 and the cardiovascular system Nat Rev Cardiol 2020 17 05 259 260 32139904 10.1038/s41569-020-0360-5 PMC7095524

[3] Evangelista A Mukherjee D Mehta R H Acute intramural hematoma of the aorta: a mystery in evolution Circulation 2005 111 08 1063 1070 15710757 10.1161/01.CIR.0000156444.26393.80

[4] Evaluation of aortic wall inflammation in acute aortic intramural hematoma using FDG-PET/CT in relation to prognostic aorta-related outcome J Nucl Med 2017 58 01 116728062597

[5] Kuehl H Eggebrecht H Boes T Detection of inflammation in patients with acute aortic syndrome: comparison of FDG-PET/CT imaging and serological markers of inflammation Heart 2008 94 11 1472 1477 18070949 10.1136/hrt.2007.127282

[6] Erbel R Aboyans V Boileau C 2014 ESC Guidelines on the diagnosis and treatment of aortic diseases: document covering acute and chronic aortic diseases of the thoracic and abdominal aorta of the adult. The task force for the diagnosis and treatment of aortic diseases of the European Society of Cardiology (ESC) Eur Heart J 2014 35 41 2873 2926 25173340 10.1093/eurheartj/ehu281

[7] Kitai T Kaji S Kim K Prognostic value of sustained elevated C-reactive protein levels in patients with acute aortic intramural hematoma J Thorac Cardiovasc Surg 2014 147 01 326 331 23219502 10.1016/j.jtcvs.2012.11.030

[8] He R Guo D C Estrera A L Characterization of the inflammatory and apoptotic cells in the aortas of patients with ascending thoracic aortic aneurysms and dissections J Thorac Cardiovasc Surg 2006 131 03 671 678 16515922 10.1016/j.jtcvs.2005.09.018

